# Mental Health Service Use by Young People: The Role of Caregiver Characteristics

**DOI:** 10.1371/journal.pone.0120004

**Published:** 2015-03-26

**Authors:** Petra C. Gronholm, Tamsin Ford, Ruth E. Roberts, Graham Thornicroft, Kristin R. Laurens, Sara Evans-Lacko

**Affiliations:** 1 Health Service and Population Research Department, King’s College London, Institute of Psychiatry, Psychology and Neuroscience, London, United Kingdom; 2 Institute of Health Research, University of Exeter Medical School, Exeter, United Kingdom; 3 Department of Forensic and Neurodevelopmental Science, King’s College London, Institute of Psychiatry, Psychology and Neuroscience, London, United Kingdom; 4 School of Psychiatry, University of New South Wales, Sydney, Australia; 5 Schizophrenia Research Institute, Sydney, Australia; The George Institute for Global Health, INDIA

## Abstract

**Aims:**

Many children and adolescents experiencing mental health problems do not receive appropriate care. Strategies to encourage appropriate access to services might be improved by a more detailed understanding of service use determinants within this group. In view of caregivers’ key role in young people’s pathways to care, this study aimed to advance understanding of caregiver-related characteristics that influence service use among young people.

**Methods:**

We interviewed 407 primary caregivers of young people aged 9-18 years, recruited from a Greater London (United Kingdom) community sample. Caregivers reported on young people’s service use in health care sector and/or education settings, and caregivers’ intended stigmatising behaviours, help-seeking attitudes, and personal service use. Logistic regression analyses examined the relationship between these caregiver characteristics and young people’s service use, controlling for young people’s clinical and socio-demographic factors.

**Results:**

Caregivers’ intended stigmatising behaviours in particular exerted a strong influence on young people’s service use within each service setting. The impact of this characteristic interacted with caregivers’ service use in influencing young people’s service use across health care and education settings and health care settings specifically. For young people’s service use within education settings, caregivers’ intended stigmatising behaviours score had a main effect.

**Conclusions:**

This study highlights the key role caregivers’ attitudes and experiences hold in young people’s service use. The findings indicate that strategies aiming to bridge the gap between young people’s service needs and utilisation might be improved by targeting stigma amongst caregivers.

## Introduction

Around one in ten children and adolescents experience mental health difficulties [1,2]. However, rates of service use amongst young people with clinically impairing levels of psychopathology are low [3], indicating a striking gap between mental health service needs and utilisation. Timely access to services is important in view of the potential long-term negative consequences of untreated early difficulties [4]. Indeed, the origin of many adult psychiatric disorders can be traced to childhood and adolescence [5] and difficulties at an early age are considered an important target for intervention efforts aiming to reduce the persistence of these problems [6]. A lack of early and effective treatment is likely to also have economic implications [7]. A detailed knowledge of the influences that underlie young people’s service contacts could enable better identification of steps that could be taken to encourage timely access to appropriate help among young people [8].

Young people’s service use is influenced by their socio-demographic and clinical characteristics [9]. Such individual characteristics alone, however, cannot fully explain service use [10,11]. Caregivers, alongside other key adult individuals, play a central role in mediating young people’s service contact [12]. Broad caregiver-related socio-demographic characteristics, as well as factors such as caregivers’ recognition of children’s problems [13–15] and family burden [16], have been associated with young people’s service use.

Further, caregiver-influences in terms of attitudes and experiences are suggested to underlie how help-seeking for their child is approached [17], including caregivers’ stigma-related beliefs and behaviours. It is likely that caregivers will encounter stigma-related barriers when seeking help for their child [18,19]; concerns around stigma and labelling in response to their child’s illness may influence help-seeking [20–22] and influence caregivers’ perceptions of treatment options [23]. Overall, however, the role of stigma in caregivers’ help-seeking remains poorly understood [21]. Caregivers’ attitudes towards treatment are also influential, with negative attitudes towards young people’s mental health services a common caregiver-reported barrier to service utilisation [10,24]. However, others report a lack of association between caregivers’ treatment attitudes and young people’s service use [25].

Furthermore, personal service use might provide caregivers with awareness of service options, which could facilitate help-seeking for their child [26]. However, only a small number of studies have explored the role of this caregiver characteristic. One study in a sample of children recruited from clinical services reported that caregivers’ history of mental health service use was not associated with the intensity or duration of help-seeking for the child [27]. Amongst a smaller sample recruited from the community, however, children’s use of mental health services was predicted by caregivers’ service utilisation [8]. Further research would help to better understand the role of this caregiver characteristic.

The existing evidence base regarding the influence of caregiver characteristics on young people’s mental health service use is characterised by several limitations. Whereas the influences of some broad caregiver influences have been explored within representative community cohorts [13,14,16], studies assessing the role of caregivers’ attitudes and beliefs typically involve clinical populations [10,20,21,24,25]. This is an important bias, as it excludes families deterred from seeking help or who did not see mental health service input as useful or necessary [28]. The perspectives of individuals in this “service gap” might provide valuable information regarding barriers to care [29]. Where the influence of caregivers’ attitudes and beliefs has been explored within non-clinical populations, limitations have been introduced through small, non-representative samples [8].

Another limitation of current studies exploring the role of caregiver characteristics conceptualisations of young people’s service use are that they are typically limited to recruitment of participants from only a single setting (e.g. [25]). However, services for young people are provided through multiple settings [1,9,30,31]. Caregivers’ involvement appears critical for services provided within health care settings [32,33], whereas in education settings individuals such as teachers also play a key role [26,34]. Caregiver characteristics could thus be expected to influence young people’s service access within the health care sector specifically, but without studies considering multiple service settings this has remained unexplored.

This study extends past research examining the role of caregiver characteristics on young people’s service use by exploring how caregivers’ public stigma and attitudes towards obtaining mental health services for themselves are associated with young people’s service use. Also, the role of caregivers’ personal service use is investigated. This study overcomes limitations of past research by exploring the influence of these caregiver characteristics from a community perspective, and considers young people’s service use in different settings. It is hypothesised that increased likelihood of young people’s service use across settings will be predicted by less caregiver-reported intended stigmatising behaviours, more positive attitudes towards formal help-seeking, and by caregivers’ own experience of service use. Secondly, it is anticipated that caregiver characteristics will be more influential in predicting service use within the health care than education sector.

## Method

### Participants

This cross-sectional study of young people aged 9 to 18 years was nested within an ongoing prospective longitudinal investigation of children recruited from the community via convenience sampling when aged 9 to 12 years; described in detail previously [35,36]. The sample was recruited from primary schools in Greater London, with the majority of participants drawn from deprived inner-city areas [37]. This sample comprises 850 caregiver-child dyads who provided contact information and consent to be re-contacted for future research following initial questionnaire screening for mental health problems during 2005–2010. This group forms the community-cohort of the Child Health and Development Study. When the current study was initiated in 2011, we retained valid contact information for 573 of these families (67.4% of the original cohort). Of these, 407 (71.0%) caregivers agreed to participate (166 caregivers/29.0% declined participation). Table 1 provides socio-demographic details of the 407 young people for whom caregivers reported service use information.

**Table 1:** Descriptive characteristics of young people and their primary caregivers (n = 407).

		*mean*	*(SD)*	*n*	*(%)*	*Missing (%)*
**Young person characteristics**	**Age in years** ^✦^	13.1	(1.7)			-
	**Sex**					-
	Female			223	(54.8)	
	Male			184	(45.2)	
	**Ethnicity**					4 (0.1)
	White			239	(58.7)	
	Black African or African-Caribbean*			*116*	(28.5)	
	South Asian or Oriental*			*28*	(6.9)	
	Other*			*20*	(4.9)	
	*Combined			164	(40.3)	
	**Socio-economic disadvantage**					1 (0.2)
	Yes			45	(11.1)	
	No			361	(88.7)	
	**Psychopathology**					1 (0.2)
	Total difficulties score (max. = 40)	11.8	(5.6)			
**Caregiver characteristics**	**Stigmatising behaviours** (max. = 20)	17.1	(2.9)			-
	**Help-seeking attitudes** (max. = 12)	9.6	(1.8)			-
	**Caregiver service use**					-
	Yes			213	(52.3)	
	No			194	(47.7)	

^✦^Age at the start of the time-period during which service use was considered.

Bivariate analyses were carried out to assess the extent to which the current study sample was representative of the community cohort from which they were recruited. No differences were observed for age or gender, however compared to the remaining community cohort (i.e., those without valid contact details and/or those who declined participation), participants in the current study reported lower psychopathology scores (t = 2.86, df = 846, p =. 004) and more frequently reported their ethnicity as white (X^2^(1) = 33.61, p≤0.01).

### Procedure

Caregivers provided information on young people’s service use, and young people’s and caregivers’ characteristics during a telephone interview. These data were linked with demographic details and ratings of child psychopathology gathered via the initial classroom questionnaires. The average time lapsing between the questionnaire screening and the time-frame considered for service use by this study was 2.8 years (SD = 1.4).

### Ethics statement

Caregivers and young people provided written informed consent for caregivers’ participation in the study. Ethical permission for the study was granted by the King’s College London Research Ethics Committee (reference PNM/10/11–6).

### Measures

#### Dependent variables: Young people’s service use

*Young people’s service use* was the main outcome of interest. The parent-report Service Assessment for Children and Adolescents (SACA; [38]) was used to derive three dichotomised past-year ratings (yes/no); service use across both health care and education settings overall, and within health care and education settings separately. The SACA assesses caregivers’ past-year experiences of accessing care for their child through inpatient and outpatient health services, and school-based services. The parent-report SACA is a valid measure of young people’s service use (kappa = 0.76; [39]) with test-retest reliability for past-year reports (ranging from 0.75 to 0.86; [40]). A subset of services assessed by the SACA were used in this study: fifteen services representing care obtained via the health care sector, and four representing care obtained via the education sector (see Table 2 for further detail).

**Table 2:** Past-year service use by sectors for the total sample and by Strengths and Difficulties Questionnaire (SDQ) score range.

	Total sample		SDQ total difficulties score range					
			Abnormal		Borderline		Normal	
	n = 407		n = 37 (9.1%)		n = 55 (13.5%)		n = 314 (77.1%)	
*Service sector*	*n*	*(%)*	*n*	*(%)*	*n*	*(%)*	*n*	*(%)*
**Health care and education**	78	(19.2)	11	(29.7)	13	(23.6)	53	(16.9)
**Health care** ▴	51	(12.5)	5	(13.5)	9	(16.3)	37	(11.8)
**Education** ^✦^	48	(11.8)	7	(18.9)	8	(14.5)	31	(9.9)

^▴^Services obtained via psychiatric hospitals, psychiatric units in general hospitals, community mental health centres or other outpatient mental health clinics, partial hospitalisations or day treatment programmes, in-home therapists or counsellors or family preservation workers, emergency rooms, paediatricians or family doctors, or professionals like psychologist, psychiatrist, social worker, marriage or family counsellor.

^✦^Services obtained via special schools, special classrooms, help in the regular classroom, or counselling in school.

#### Independent variables

(a) Caregiver characteristics

*Caregivers’ intended stigma-related behaviours* were assessed using the Reported and Intended Behaviour Scale (RIBS; [41]). Responses to four items assessing future intentions to live with, work with, live nearby, and continue a relationship with a person affected by mental illness are summed into a composite score; higher total scores reflect less intended stigmatising or discriminatory behaviours towards people with mental illness. This measure has moderate test-retest reliability of 0.75, and good internal consistency; α = 0.85 [41]. In this study, a comparable internal consistency rating was observed (α = 0.71).

*Caregivers’ attitudes towards seeking professional mental health services* were assessed using three items sourced from the National Comorbidity Survey Replication [42], considering willingness, comfort, and perceived stigma associated with seeking professional help for an emotional problem. Responses were summed to a composite score; higher total scores indicate more positive help seeking attitudes. This rating is considered useful to assess overall attitudes towards professional mental health services despite reports of low internal consistency on this measure [43]. In this study, internal consistency was α = 0.51.

*Caregivers’ use of mental health services* was assessed through the question “Have you ever spoken to, for instance, a general practitioner or family doctor, a psychological therapist/counsellor or other source of help on your own behalf, either in person or by telephone, about being anxious or depressed, or due to any other mental, nervous or emotional problem?” sourced from the South East London Community Health study [44]. Responses were dichotomised (service use/no service use).

(b) Young person characteristics

*Socio-demographic characteristics*. Young people were characterised in terms of sex and age at beginning of the 12-month period in which young people’s service use was quantified. Caregivers reported on the young people’s ethnicity in accordance with the 2001 UK Census ethnic categories, from which ethnic groups were subsequently amalgamated into the following categories: white, black African or African-Caribbean (including mixed with white), Asian or Chinese (including “Asian, other”, and mixed with white), and other ethnicity. For inferential analyses ethnicity was considered as a dichotomised measure (white/other). The child’s caregiver-reported eligibility to receive free school meals (yes/no) was used as an indicator of family socio-economic disadvantage [45].

*Clinical characteristics*. Child psychopathology was assessed through the child-report Strengths and Difficulties Questionnaire (SDQ; [46]). The internal reliability, test-retest reliability, and validity for the child-report version of the SDQ are satisfactory [47]. The measure yields a Total Difficulties score, reflecting the sum of difficulties on the psychopathology subscales of emotional symptoms, conduct problems, hyperactivity-inattention, and peer relationship problems); higher scores indicate a greater level of difficulties. This score is considered a sound measure of overall child mental health problems [48], and was used for analysis in this study.

### Analyses

Data were analysed using IBM SPSS Statistics 20 for Windows.

Preliminary bivariate analyses revealed associations between the independent variables; to increase interpretability of the results, continuous variables were mean-centered [49]. Outliers were investigated through visual displays of the data, and the standard errors for the independent variables in the final regression models indicated no evidence of multicollinearity.

Analysis of the data proceeded first with examination of unadjusted associations between the independent and outcome variables. Following this, multivariable logistic regression analyses examined the strength of association between caregiver characteristics and young people’s service use (i) across health care and education sectors, (ii) within the health care sector only, and (iii) within the education sector only, controlling for the influence of young people’s characteristics. Variables were entered in blocks, to capture whether adding caregiver characteristics to the multivariable models provided an extended understanding of young people’s service use influences. Where significant interaction terms between independent variables were observed, these were added as a final block in the analyses and explored further in stratified analyses. For all models, the odds ratios (and 95% confidence intervals) are given. The Cox & Snell and Nagelkerke’s R-squared values before and after the addition of caregiver characteristics are reported, to reflect associated increases in the variance explained by the model.

Subsequent analyses explored interaction effects between independent variables.

## Results

### Sample

Summary statistics characterising young people’s socio-demographic and clinical features and caregiver characteristics are presented in Table 1. Young people’s past-year rates of service use are reported in Table 2 for the entire sample, and separately for those reporting child psychopathology in the abnormal (~top 10^th^ percentile), borderline (~top 20–10^th^ percentile) and normal (~0–80^th^ percentile) ranges defined for the SDQ in the UK population (www.sdqinfo.org).

### Inferential analyses

#### (i) Overall health care and education sector service use

Table 3 shows the associations between the independent variables and young people’s overall service use. In the final adjusted model, disadvantaged young people (i.e., those eligible for free school meals) were more likely to have used services than more advantaged young people. Amongst young people whose caregivers had not used services, caregivers’ higher RIBS scores (i.e., less intended stigmatising behaviours) were associated with an increased likelihood of service use. Within this final model, more positive attitudes towards help-seeking amongst caregivers were associated with a decreased likelihood of mental health service use. The Cox & Snell and Nagelkerke *R*-squared statistics increased from 3.4–5.4% to 7.2–11.6% with the addition of caregiver characteristics to the model.

**Table 3:** Unadjusted and adjusted odds ratios (ORs) and 95% confidence intervals (CIs) for factors associated with young people’s service use across health care and education settings.

*Variable*			*Unadjusted model; OR (95% CI)*		*Adjusted model; OR (95% CI)*							
					*Block 1*		*Block 2*		*Block 3*		*Final model*	
**Demographic characteristics**	Age		1.0	(0.9–1.2)	1.0	(0.9–1.2)	1.0	(0.9–1.2)	1.0	(0.9–1.2)	1.0	(0.9–1.2)
	Sex	Female	1.0		1.0		1.0		1.0		1.0	
		Male	1.7	(1.1–2.9)*	1.7	(1.0–2.9)*	1.6	(1.0–2.7)▴	1.6	(1.0–2.8)▴	1.6	(0.9–2.7)▴
	Ethnicity	White	1.0		1.0		1.0		1.0		1.0	
		Other	1.2	(0.8–2.0)	1.1	(0.7–1.9)	1.1	(0.6–1.8)	1.4	(0.8–2.4)	1.4	(0.8–2.4)
	Socio-economic disadvantage	Yes	1.0		1.0		1.0		1.0		1.0	
		No	0.4	(0.2–0.8)*	0.5	(0.2–1.0)*	0.4	(0.2–0.9)*	0.4	(0.2–0.9)*	0.4	(0.2–0.9)*
**Clinical characteristics**	Total difficulties		1.1	(1.0–1.1)*			1.0	(1.0–1.1)*	1.0	(1.0–1.1)▴	1.0	(1.0–1.1)
**Caregiver characteristics**	Stigmatising behaviours (RIBS)		1.1	(1.0–1.2)*					1.2	(1.0–1.3)**	1.0	(0.9–1.2)
	Help-seeking attitudes		0.9	(0.8–1.0)					0.9	(0.7–1.0)▴	0.8	(0.7–1.0)*
	Caregiver service use (CSU)	Yes	1.0						1.0		1.0	
		No	0.8	(0.5–1.3)					0.8	(0.4–1.3)	0.6	(0.4–1.1)
	RIBS*CSU interaction	Yes CSU									1.0	
		No CSU									1.3	(1.1–1.7)*

* p≤0.05;

** p≤0.01;

^▴^p<0.10.

#### (ii) Health care sector service use only

The findings regarding young people’s likelihood of service use within the health care sector are presented in Table 4. The model failed to demonstrate an improvement over the null model when the main effects of all independent variables were included. However, the addition of the interaction term between caregivers’ service use and intended stigma-related behaviours yielded a statistically significant improvement to the model (x^2^ = 6.58, df = 1, p = 0.010), with the final model including this interaction term approaching statistical significance (x^2^ = 16.53, df = 9, p = 0.057). Within this model, only the interaction between caregivers’ service use and stigma-related behaviours score was significantly associated with the likelihood of young people’s service use. That is, for young people whose caregivers had not themselves used services, caregivers’ higher RIBS scores (indicating less intended stigmatising behaviours) were associated with increased likelihood of young people’s service use. Cox & Snell and Nagelkerke *R*-squared statistics indicated that this final model explained between 4.0–7.6% of the variance in the data.

**Table 4:** Unadjusted and adjusted odds ratios (ORs) and 95% confidence intervals (CIs) for factors associated with young people’s service use within health care settings.

*Variable*				*Unadjusted model; OR (95% CI)*		*Adjusted model; OR (95% CI)*							
						*Block 1*		*Block 2*		*Block 3*		*Final model*	
**Demographic characteristics**	Age			1.0	(0.9–1.2)	1.0	(0.9–1.2)	1.0	(0.9–1.2)	1.0	(0.9–1.2)	1.0	(0.9–1.2)
	Sex	Female		1.0		1.0		1.0		1.0		1.0	
		Male		1.6	(0.9–2.8)	1.6	(0.9–2.9)	1.5	(0.8–2.8)	1.6	(0.8–2.9)	1.5	(0.8–2.8)
	Ethnicity	White		1.0		1.0		1.0		1.0		1.0	
		Other		1.5	(0.8–2.7)	1.3	(0.7–2.5)	1.3	(0.7–2.4)	1.5	(0.8–2.9)	1.5	(0.8–3.0)
	Socio-economic disadvantage	Yes		1.0		1.0		1.0		1.0		1.0	
		No		0.5	(0.2–1.2)	0.6	(0.2–1.3)	0.5	(0.2–1.3)	0.5	(0.2–1.3)	0.6	(0.2–1.4)
**Clinical characteristics**	Total difficulties			1.0	(1.0–1.1)			1.0	(1.0–1.1)	1.0	(1.0–1.1)	1.0	(1.0–1.1)
**Caregiver characteristics**	Stigmatising behaviours (RIBS)			1.1	(1.0–1.2)					1.1	(1.0–1.2)	1.0	(0.8–1.1)
	Help-seeking attitudes			1.0	(0.8–1.2)					0.9	(0.8–1.1)	0.9	(0.8–1.1)
	Caregiver service use (CSU)	Yes		1.0						1.0		1.0	
		No		0.8	(0.5–1.3)					0.8	(0.4–1.5)	0.7	(0.3–1.3)
	RIBS*CSU interaction	Yes CSU										1.0	
		No CSU										1.4	(1.1–1.8)*

*p≤0.05.

#### (iii) Education sector service use only

Table 5 displays associations between the independent variables and young people’s service use within the education sector. In the final adjusted model, socioeconomic disadvantage (compared to greater advantage) and caregivers RIBS scores indicative of less intended stigmatising behaviours were associated with an increased likelihood of young people’s service use. No significant interaction terms were observed within this model. The Cox & Snell and Nagelkerke *R*-squared statistics indicated that, compared to the model including young people characteristics only, when caregiver characteristics were added, the variance in the final model increased from 3.9–7.5% to 5.9–11.4%.

**Table 5:** Unadjusted and adjusted odds ratios (ORs) and 95% confidence intervals (CIs) for factors associated with young people’s service use within education settings.

*Variable*				*Unadjusted model; OR (95% CI)*		*Adjusted model; OR (95% CI)*					
						*Block 1*		*Block 2*		*Final model*	
**Demographic characteristics**	Age			0.9	(0.8–1.1)	0.9	(0.8–1.1)	0.9	(0.8–1.1)	0.9	(0.8–1.1)
	Sex	Female		1.0		1.0		1.0		1.0	
		Male		2.0	(1.1–3.7)*	1.9	(1.0–3.6)*	1.7	(0.9–3.3)▴	1.8	(0.9–3.4)▴
	Ethnicity	White		1.0		1.0		1.0		1.0	
		Other		1.0	(0.6–1.9)	0.9	(0.5–1.7)	0.8	(0.4–1.6)	1.0	(0.5–2.0)
	Socio-economic disadvantage	Yes		1.0		1.0		1.0		1.0	
		No		0.3	(0.1–0.6)**	0.3	(0.1–0.7)**	0.3	(0.1–0.7)**	0.2	(0.1–0.6)***
**Clinical characteristics**	Total difficulties			1.1	(1.0–1.1)▴			1.1	(1.0–1.1)▴	1.0	(1.0–1.1)
**Caregiver characteristics**	Stigmatising behaviours			1.2	(1.0–1.3)*					1.2	(1.0–1.4)*
	Help-seeking attitudes			0.9	(0.8–1.1)					0.9	(0.7–1.0)
	Caregiver service use	Yes		1.0						1.0	
		No		0.9	(0.5–1.7)					1.1	(0.6–2.1)

*p≤0.05;

**p≤0.01;

***p≤0.001;

^▴^p<0.10.

Table 6 provides a summarised overview of the results of the final adjusted models of these three inferential analyses.

**Table 6:** Overview of the results of the final adjusted models for each of the three service use outcomes assessed in this study.

*Variable*		Overall; health care and education sector	Health care sector only	Education sector only
**Demographic characteristics**	Age	-	-	-
	Sex (“Male”; reference “Female”)	↑▲	-	↑▲
	Ethnicity (“Other”; reference “White”)	-	-	-
	Socio-economic disadvantage (“Yes”; reference “No”)	↑ ★	-	↑ ★
**Clinical characteristics**	Total difficulties	-	-	-
**Caregiver characteristics**	Stigmatising behaviours; RIBS	-	-	↑ ★
	Help-seeking attitudes	↓★	-	-
	Caregiver service use; CSU (“No”, reference “Yes”)	-	-	-
	RIBS*CSU interaction (“No CSU”; reference “Yes CSU”)	↑ ★	↑ ★	-

★ p≤0.05;

^▲^ p<0.10

^-^ non-significant association

^↑/↓^ direction of association; increased/decreased likelihood of service use.

As the lapse in time between when data on psychopathology and service use were collected might contribute to the finding of young people’s difficulties having little influence on likelihood of service contact, this relationship was explored by repeating analyses with the inclusion of an additional variable reflecting the elapsed time, and an interaction term between this and psychopathology scores. This interaction term was, however, not significant (overall service use: OR = 0.995, 95% CI 0.96–1.03, p = 0.754; health care sector; OR = 0.995, 95% CI 0.96–1.03, p = 0.787; education sector: OR = 0.993, 95% CI 0.95–1.03, p = 0.737), and controlling for this did not change the outcomes of the analyses.

An additional exploration was also carried out to examine the role of another indicator of families’ socio-economic status: caregivers’ education level (at least one caregiver having university level education; yes vs. no). This variable was associated with young people’s service contact in education settings only, whereas the indicator of families’ socio-economic disadvantage through eligibility for free school meals was associated with young people’s overall service use as well as service contact in education settings specifically.

### Stratified analyses

Models with significant interaction effects between caregivers’ service use and stigma-related characteristics were repeated, stratified by caregivers’ service use.

### No caregiver service use

#### (i) Overall health care and education sector service use

Increased likelihood of service use was associated with male gender (relative to females; OR = 2.64, 95% CI 1.13–6.18, p = 0.025), socioeconomic disadvantage (compared to greater advantage; OR = 0.13, 95% CI 0.03–0.57, p = 0.007), reductions in caregivers’ help-seeking attitudes scores (OR = 0.79, 95% CI 0.63–1.00, p = 0.045), and caregivers RIBS scores indicative of less intended stigmatising behaviours (OR = 1.39, 95% CI 1.13–1.71, p = 0.002).

#### (ii) Health care sector service use

The only variable associated with young people’s increased likelihood of service use was caregivers’ RIBS scores indicative of less intended stigmatising behaviours (OR = 1.35, 95% CI 1.08–1.68, p = 0.009).

### Caregiver service use

Both models considering young people’s likelihood of service use (i) across sectors and (ii) within the health sector specifically failed to predict young people’s service use better than the null model.

## Discussion

This study aimed to advance the understanding of the role of caregiver characteristics in young people’s mental health service use. Caregiver characteristics were found to exert a key influence on young people’s likelihood of service use, albeit not entirely as hypothesised.

When caregivers’ stigma-related characteristics was considered as a main effect, as hypothesised, young people’s likelihood of service use across health and education settings increased as caregivers reported less intended stigmatising behaviours. This concurs with previously theorised patters (e.g. [18]). However, a more complex picture emerged when considering interactions between caregiver characteristics. Caregivers’ stigma influenced young people’s contact with health care sector services, and overall service use within health and education settings, only amongst those young people whose caregivers had not used mental health services. For services provided within education settings no such interaction was observed; caregivers’ stigma-related characteristics influenced service use for all young people irrespective of caregivers’ own service use. These findings highlight how the influence of caregivers’ stigma on young people’s service use might not be fully understood without also considering the effect of other caregiver characteristics and the service setting. Unlike hypothesised, no main effect was observed for caregivers’ personal service contact, which exerted an influence only via the interaction with caregivers’ stigma.

In this study, caregivers’ stigma-related characteristics were not associated with young people’s services provided wholly or partially within health care services when caregivers had experience of service use themselves. This may relate to with the finding that less stigma towards child mental health services was expressed by parents who had previously used mental health services [8]. Also, caregivers’ personal service use might have provided a sense of familiarity with health service settings, which would correspond with how stigma is best overcome through personal exposure and achieving familiarity with the stigmatised entity [50]. That no comparable influence of caregivers’ service use on stigma was observed in relation to young people’s education sector service use could be because caregivers’ service use is likely to facilitate an awareness of health services exclusively, as education setting services cater predominantly for young people [34]. Also, the influence of other ‘gatekeepers’ or significant peer influence may be more important for determining service use in education settings.

Contrary to our predictions, more positive help-seeking attitudes by caregivers were associated with decreased likelihood of young people’s service use. Preliminary bivariate analyses between the independent variables showed, as might be intuitively expected, a relationship between caregivers’ own service use and more positive help-seeking attitudes. As such, our findings indicate that it might be misleading to assume that caregivers’ help-seeking attitudes relate to their children’s service use in a directly comparable manner to their own service contact.

As hypothesised, caregiver characteristics appeared more influential for young people’s service use within health care than education settings. Within education settings, caregivers’ stigma influenced young people’s service use, whereas in the health settings both caregivers’ stigma and service use were involved. These findings are in line with how, besides parents, other individuals such as teachers are likely to influence young people’s service use within education settings [26,34]. Young people might also have more autonomy to access services within school settings. This difference also highlights that although certain caregiver factors might impede young people’s access to health care services, they might not pose an equivalent barrier to services within education settings. More research is needed to explore this further.

In these data we did not identify an association between the service use outcomes and young people’s age or ethnicity. It is however possible that in a sample with a wider age range or a broader ethnic sample these variables would also have been found to influence young people’s likelihood of service contact.

Overall, this study illustrates the important role caregiver-related influences have in young people’s service use. The finding that caregivers’ public stigma was associated with young people’s service use within this community cohort extends past literature where caregivers’ stigma has been explored primarily within clinical samples (e.g. [21,24]). Furthermore, young people’s prior psychopathology was not associated with overall service use once the influence of caregiver characteristics was considered. This finding of caregiver characteristics appearing more influential than psychopathology scores in predicting young people’s likelihood of service contact concurs with previous reports of caregivers’ attitudes towards mental health services [10] and strain [16] predicting young people’s service use more strongly than child psychopathology.

These results also suggest that caregiver characteristics influence young people’s service use in a complex manner, and future research drawing on qualitative and / or mixed methods could provide further insights regarding how stigma-related factors and caregivers’ experiences and beliefs can influence service utilisation amongst children and adolescents.

### Strengths and limitations of the study

This study extends previous literature that explores young people’s service use by analysing the role of, and interactions between, multiple caregiver characteristics beyond those directly related to young people’s mental health or service use. By examining these influences within a community sample, this study could consider access to services within both health and education sectors and, critically, was not limited to individuals who had successfully established service contact or met diagnostic criteria. However, due to the community nature of the sample not all participants had used services, which meant that we had limited power to investigate type of service use in greater detail (e.g., within specific settings). In relation to service use, it is also necessary to recognise that a service use rating based on clinical or administrative records in addition to caregiver reports could have provided a more comprehensive picture of service contact, especially in relation to education sector service use [39]. A further limitation is that scores on young people’s psychopathology were collected on average 2.8 years prior to the current study. More recent psychopathology scores might have constituted a stronger service use influence. However, the impact of this time-lapse was explored, and adding this variable to our models did not influence the outcomes. Also, the model assessing service use within health care settings was only marginally significant (p =. 057). However, the interpretation that young people’s service use within this setting was explained by the interaction between caregivers’ stigma and service use is considered justified: in the stratified analyses, stigma was a significant predictor within an overall significant model when caregivers had not used services. Finally, it is necessary to recognise that our study sample was not fully representative of the original community cohort from which it was drawn, and the experiences of young people with higher psychopathology scores or who reported their ethnicity as other than white might be underrepresented. Furthermore, it is possible that there are other characteristics, for which we held no data and could as such not explore, which are present in our data in a non-representative manner. However, even if there are possible demographic or motivational aspects in which our study sample differs from the original study cohort or community at large, our findings still provide an important indication of how caregivers’ characteristics might influence young people’s service use and how key influences such as caregivers’ stigma warrant to be recognised in further studies.

### Implications

This study highlights the key role caregiver-related factors hold in young people’s mental health service use. Comprehensive strategies recognising caregivers’ involvement seem crucial to reduce young people’s underuse of services. In particular, caregivers’ stigma-related characteristics could constitute a key target for such efforts, as their intended stigmatising behaviours influenced young people’s service use to some extent within all service settings considered in this study. This corresponds with the pervasive manner in which stigma can form a barrier for adults’ service use [51–53]. Facilitating a sense of familiarity with and awareness of service options amongst caregivers might help to bridge the gap between young people’s needs and utilisation of mental health services.

### Conclusion

This study advances our understanding regarding influences underlying young people’s service use through a focus on multiple caregiver characteristics and their interactions, and young people’s services provided within multiple settings. To our knowledge, this is the first study to provide such a detailed exploration of the role of caregiver characteristics in relation to service use among a community sample of young people. Our findings suggest that caregivers’ attitudes and experiences, in particular stigma-related characteristics, influence service use amongst children and adolescents.
